# Data integration and mechanistic modelling for breast cancer biology: Current state and future directions

**DOI:** 10.1016/j.coemr.2022.100350

**Published:** 2022-06

**Authors:** Hanyi Mo, Rainer Breitling, Chiara Francavilla, Jean-Marc Schwartz

**Affiliations:** 1 Division of Evolution, Infection and Genomics, School of Biological Sciences, University of Manchester, Manchester, M13 9PT, UK; 2 Manchester Institute of Biotechnology, School of Natural Sciences, University of Manchester, Manchester, M1 7DN, UK; 3 Division of Molecular and Cellular Function, School of Biological Sciences, University of Manchester, Manchester, M13 9PT, UK; 4 Manchester Breast Centre, Manchester Cancer Research Centre, University of Manchester, M13 9PT, Manchester, UK

**Keywords:** Breast cancer, Precision oncology, Multi-omics modelling, Deep learning, Network biology

## Abstract

Breast cancer is one of the most common cancers threatening women worldwide. A limited number of available treatment options, frequent recurrence, and drug resistance exacerbate the prognosis of breast cancer patients. Thus, there is an urgent need for methods to investigate novel treatment options, while taking into account the vast molecular heterogeneity of breast cancer. Recent advances in molecular profiling technologies, including genomics, epigenomics, transcriptomics, proteomics and metabolomics data, enable approaching breast cancer biology at multiple levels of omics interaction networks. Systems biology approaches, including computational inference of ‘big data’ and mechanistic modelling of specific pathways, are emerging to identify potential novel combinations of breast cancer subtype signatures and more diverse targeted therapies.

## Introduction

Breast cancer is the most common malignancy threatening women's health worldwide [1]. It affects approximately 1 in 8 women over the course of their lifetime and is also sometimes seen in men, where malignant lesions can occur to ducts in the retro-areolar area, although with much lower incidence [1]. Breast cancer treatments include surgery and radiotherapy to treat early-stage patients with non-metastatic disease, often in combination with adjuvant/neoadjuvant therapy to prevent recurrence [2,3]. Neoadjuvant therapies deliver chemotherapy (e.g., pertuzumab and trastuzumab) or hormone therapy (e.g., aromatase inhibitor) to reduce the size of tumour before breast-conserving surgery [3,4]. Endocrine therapies, chemotherapies and targeted drugs (e.g., cyclin-dependent kinase [CDK]4/6 inhibitors), are the most widely used for advanced, metastatic patients [2, 3, 4].

With the advent of precision oncology [5], the molecular characteristics of an individual's tumour can be targeted in a specific manner. Targeted therapies for breast cancer are so far based only on the status of hormone receptors (HRs) and human epidermal growth factor receptor 2 (HER2): oestrogen and progesterone receptor-positive (ER/PR+) patients are usually treated with endocrine therapies (e.g., tamoxifen), while HER2+ patients are treated with anti-HER2 target therapies (e.g., trastuzumab) [2]. For patients with triple-negative breast cancer (TNBC), without significant overexpression of any of the HRs or HER2, the expression of programmed death-ligand 1 (PD-L1) has recently been identified as a successful marker to administer immunotherapy (e.g., atezolizumab and pembrolizumab) plus chemotherapy (e.g., nab-paclitaxel) [6,7]. Despite huge research efforts towards the molecular characterisation of breast cancer over the last decades, treatment decisions are still mainly based on this limited set of biomarkers (HR, HER2), and therefore treatment strategies remain insufficiently targeted. Tumour heterogeneity and mechanisms of resistance to treatments are among the causes of inefficient treatments and tumour recurrence [8,9]. More recently, other potential targets have been discovered using pharmacogenomics approaches, which study genetic variants of individual patients by integrating omics data to predict drug responses [9]. For example, kinases CDK4/6 and phosphoinositide 3-kinase (PI3K) are reported to be effective drug targets to overcome post-treatment resistance introduced by endocrine therapies for ER + breast cancer [10,11]. These two kinase inhibitors are also potentially effective for treating TNBC in combination with other drugs, with a few ongoing clinical trials initiated (e.g., alpelisib + nab-paclitaxel for TNBC patients with a phosphatidylinositol-4,5-bisphosphate 3-kinase catalytic subunit alpha [PIK3CA] mutation) [12,13].

**Figure 1:**
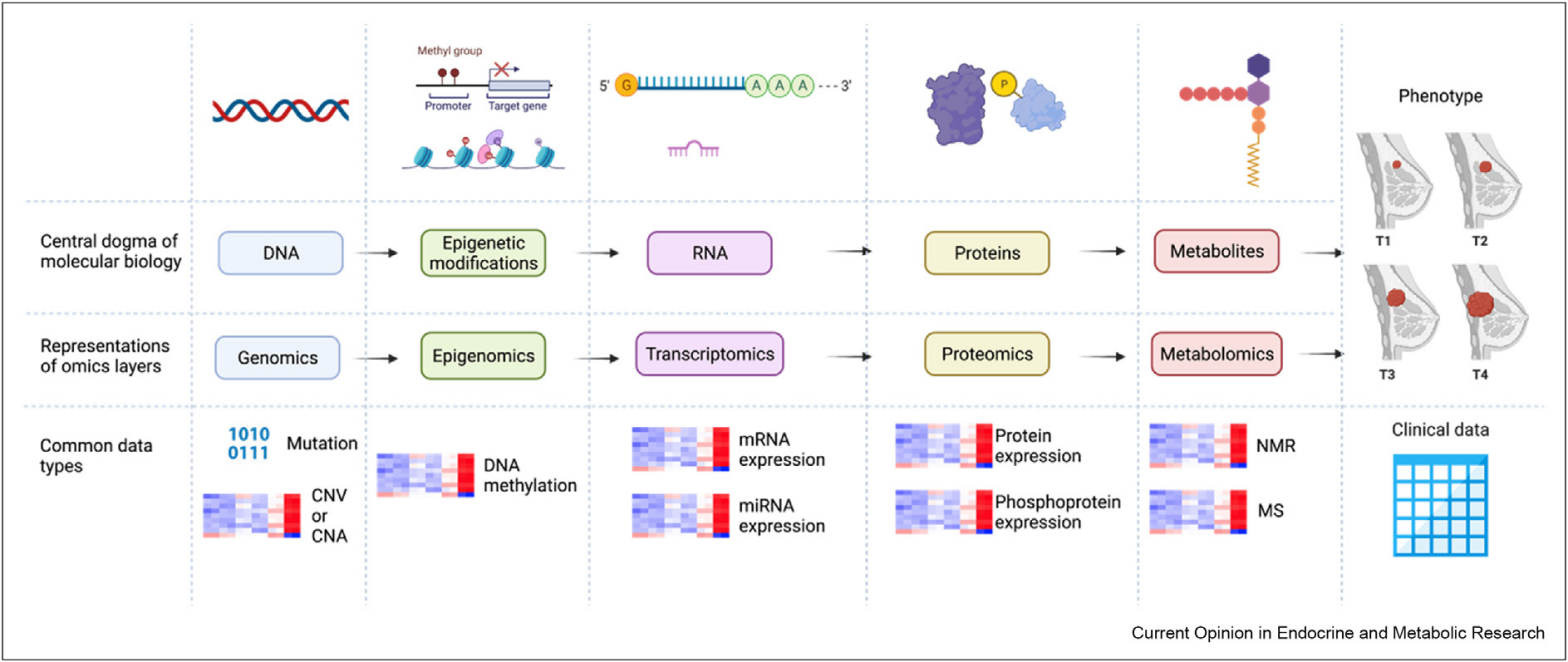
Selected Omics/clinical data commonly used in bioinformatics analysis.

Recent advances in molecular profiling technologies, including next-generation sequencing, transcriptomics and high-throughput mass spectrometry-based proteomics and metabolomics, have started to increase the number of potential targets for the development of personalised treatments [14, 15, 16]. Systems biology approaches that integrate large volumes of omics data from profiling technologies into molecular and causal networks are expected to extend the mechanistic understanding of breast cancer across all levels of the cellular hierarchy, from gene regulatory networks and signalling cascades to protein–protein interaction graphs and metabolic pathways [14,15] (Figure 1). Multi-omics integration aims at discovering novel drug targets and diagnostic biomarkers at all levels of the cellular system by establishing a personalised landscape for patient stratification, drug administration and prognosis. Network analysis is underlying most multi-omics integration in systems biology approaches, explicitly or implicitly, from statistical inference techniques to mechanistic modelling.

Here, after introducing available omics data and databases from molecular profiling technologies analysing cancer samples, including those derived specifically from breast tumour samples, we will discuss how recent developments in data integration and mechanistic modelling can be used towards the development of more efficient personalised treatments. Two main types of multi-omics approaches can be distinguished: 1) data-driven statistical inference methods, in particular the latest deep learning techniques, to predict gene features that potentially affect patient characteristics and clinical responses; and 2) molecular target-focused mechanistic network modelling methods to identify novel therapeutic options. We will show that these two approaches can identify additional biomarkers, enrich our knowledge of the network underlying breast cancer mechanisms, be used for better patient molecular subtyping and for predicting drug response and post-treatment outcomes, and narrow down novel ‘driver’ pathways at the protein and metabolite level to be validated via *in vitro* and *in vivo* models.

## Data and databases from molecular profiling technologies

For each order of the central dogma, omics data layers and type are represented. Although copy number variations (CNVs, inherited from germline) and copy number alternations (CNAs, acquired in somatic cells) are considered as mutations, they do not change the sequence and are stored and analysed as continuous data, in contrast to binary mutation data. Metabolite concentrations are mainly acquired by mass spectrometry (MS) and nuclear magnetic resonance (NMR) experiments. Clinical data attributed to patient samples commonly include age at diagnosis, ER/PR/HER2 status, and tumour grade and size. Created with BioRender.com.

Molecular profiling technologies include analyses of samples from cancer patients or from biological models of cancer at a global scale on multiple levels. In the order of the ‘central dogma’ of molecular biology, the major technologies target genetic mutations (genomics), epigenetic modifications (epigenomics), RNA expression (transcriptomics), protein abundance and post-translational modifications (proteomics) and metabolite concentrations (metabolomics) [14,15,17] (Figure 1). These technologies already successfully contribute a variety of information to improve targeted clinical approaches to breast cancer. At the genomic level, for example, people with inherited mutations of breast cancer susceptibility genes (BRCA1 and BRCA2) are recommended to have regular screening and prophylactic bilateral mastectomy [18]. Transcriptomics applications such as Prediction Analysis of Microarray 50 (PAM50), MammaPrint and Oncotype DX are used for treatment recommendations based on gene expression signatures [1]. Analyses on DNA methylation accompanied by differentially expressed genes (DEGs) have identified novel methylation markers with diagnostic and prognostic values [19]. Mass spectrometry-based proteomics assays can be assessed through a variety of samples including urine and blood, which are promising as a regular monitoring approach in future clinical practice [20]. Metabolic heterogeneity can provide novel insights about the breast tumour microenvironment in association with cancer progression, drug resistance and metastasis [21]. Data generated by these molecular profiling technologies, known as omics data, contribute to various cancer-specific data consortia, such as the International Cancer Genome Consortium (ICGC) [22] and The Cancer Genome Atlas (TCGA) [23], enabling researchers to download data and customise analytic approaches aiming at precision medicine from multi-omics integration.

Omics data should be considered to study not only interactions in the current layer (e.g., co-expression network) but also interactive effects across layers (e.g., gene regulatory networks). For example, mutation and copy number alone cannot determine mRNA expression, since DNA methylation modulates transcription. Although proteins are translated from mRNA, protein expression is also regulated by the silencing effects of miRNA including translational repression and mRNA degradation. In turn, protein products such as transcription factors affect mRNA expression throughout gene regulatory networks by binding to DNA sequences. However, integrating all omics data is challenging as these data types are unevenly deposited in publicly accessible data repositories, as shown for the cancer-specific data resources provided in Table 1. This is especially the case for proteomics and metabolomics data which are generally underrepresented for all cancer types. For instance, on the ICGC data portal, there are only 298 among all 1969 donors with protein expression data available from breast cancer projects [22]. By contrast, Clinical Proteomic Tumor Analysis Consortium (CPTAC) hosts only MS-based proteomics data including the analysis of various post-translational modifications (PTMs) such as phosphoproteome, acetylome and glycoproteome but it lacks other omics data types [24]. Although CPTAC uses TCGA samples and is integrated on the Genomic Data Commons (GDC) data portal, only 12.2% of TCGA entries have been attributed to protein expression in breast cancer so far [25]. The integration of metabolomics data with other omics data is even scarcer for cancer research as reflected by the fact that they are usually archived in separate single-omics databases such as MetaboLights [26]. Considering that metabolomics has emerged more recently than other omics technologies for breast cancer research [14,27], the relatively small number of breast cancer-related studies in MetaboLights will require future community work to enrich the representation of this important complementary omics data type.

**Table 1:** Selected data resources from molecular profiling technologies useful for breast cancer data analysis, grouped into three categories in line with their purposes and usages. Resources under ‘data portals and databases’ not only can host various cancer-specific data portals, including the selected data projects listed under the category ‘ongoing data projects’, but also provide download possibilities and other bioinformatics tools for downstream analysis such as visualisation and pathway enrichment analysis. ‘General omics data sources’ list four representative databases for gene expression, protein expression and compound information with a larger scope than cancer research. The International Cancer Genome Consortium (ICGC) [22], Genomic Data Commons (GDC) [25], cBioPortal for Cancer Genomics [57], Catalogue Of Somatic Mutations In Cancer (COSMIC) [58], Transcriptome Alterations in CanCer Omnibus (TACCO) [59], Genomics of Drug Sensitivity in Cancer (GDSC) [44]. The Cancer Genome Atlas (TCGA) [23], Clinical Proteomic Tumor Analysis Consortium (CPTAC) [24], Molecular Taxonomy of Breast Cancer International Consortium (METABRIC) [60,61], Gene Expression Omnibus (METABRIC) [62], PRoteomics IDEntifications (PRIDE) [63], MetaboLights [26]. Data types are described according to omics levels.

	Data types	Description	Highlight
Cancer-specific data portals or databases			
ICGC	G E T P C	A comprehensive interactive database portal containing data from 84 cancer programs worldwide, 77 million somatic mutations and molecular data from over 24,000 donors.	ICGC encompasses various index search technologies to optimise computational performance for large-scale searches.
GDC	G E T P C	An information web-based database harmonising data from various cancer projects including TCGA and CPTAC (see below) for visualisation and downloading.	GDC aims at developing a holistic taxonomy of cancer types and providing state-of-the-art bioinformatics tools to enhance the interpretation of data.
cBioPortal for Cancer Genomics	G E T P C	A data portal hosting data from over 5000 tumour samples from 20 cancer studies, including the METABRIC (see below) project, enabling both web access and script libraries (e.g., MATLAB and R) to meet customised analysis requirements.	The cBioPortal provides unique functionality of interactive network analysis for studying the cancer of interest and supports the visualisation of mutations within Pfam protein domains.
COSMIC	G E T C	A thorough data portal with a specialised focus on somatic mutations driving 10 cancer development, consisting of 6 million coding mutations across 1.4 million tumour samples.	COSMIC provides an improved data visualisation and downloading portal that also hosts a 3-D protein structure exploration tool (COSMIC-3D) to link mutations to protein function.
TACCO	T C	An easy-to-use interface for connecting transcriptome data (e.g., differentially expressed genes, DEGs and differentially expressed miRNAs, DEmiRNAs) and pathway dysregulations to clinical outcomes in pan-cancer studies.	TACCO allows users to either select DEGs/DEmiRNAs from pre-defined gene lists or upload genes of interest to perform downstream tasks (e.g., KEGG pathway/gene ontology enrichment analysis, multi-gene prognostic models).
GDSC	G E T C	A pharmacogenomic data repository hosting information on anti-cancer drug sensitivity and molecular markers of drug responses, containing overall 518 compounds targeting 24 pathways.	GDSC differentiates data by the response to anti-cancer drugs and by pathways in a pan-cancer and pan-drug manner. It also allows browsing data by tissue-specific terms and incorporates TCGA cancer classifications and COSMIC mutation identities.
Ongoing cancer-specific data projects			
TCGA	G E T P C	A long-term cancer genomic project launched in 2006, characterising more than 20,000 primary cancer samples and mapping them to 33 cancer types.	TCGA utilises numerous data generation platforms including RNA-seq, miRNA-seq, DNA-seq, array-based SNP, array-based DNA methylation sequencing, and reverse-phase protein array to provide a collection of omics data types for cancer studies.
CPTAC	P	A project emphasising mass spectrometry-based protein profiling of tumour samples in accordance with TCGA projects.	CPTAC incorporates the CPTAC Common Data Analysis Platform (CDAP) to diminish instrumentation variability among data and to better integrate with TCGA datasets.
METABRIC	G T C	A BC-specific data program for elucidating molecular drivers with an extensive focus on inherited copy number variations/acquired copy number alterations (CNVs/CNAs).	METABRIC identifies novel loci that contribute to breast carcinogenesis and discovers that somatic CNAs show more prognostic power in a long-term clinical context compared to germline CNVs.
General single-omics data sources			
GEO	G E T	A public archive for researchers to submit array- or sequence-based functional genomic data, accessible by both web portal and R library interface.	GEO contains almost 147,000 breast cancer-related studies and 184 breast cancer-related datasets to date.
ArrayExpress	G E T P M	A public archive hosting data generated by a variety of profiling technologies with most on DNA, RNA assays while few on protein and metabolic profiling.	ArrayExpress contains over 4000 experiments regarding breast cancer including 788 DNA, 3314 RNA and 29 protein assays.
PRIDE	P	A proteome-focused repository mainly for depositing mass spectrometry-based proteomics data, including protein and post-translational modification expression data.	PRIDE hosts over 500 breast cancer proteomics datasets to date with details on sample preparation and data processing.
MetaboLights	M	A database hosting metabolomics experimental data, relevant information and a central hub for metabolomics related data and tools.	MetaboLights encompasses over 200 breast cancer-related compounds and around 134 case studies for breast cancer research.

G, genomics; T, transcriptomics; E, epigenomics; P, proteomics; M, metabolomics; C, clinical data

As proteomics and metabolomics data are not as abundant as other data layers (Figure 1), current discoveries often first hypothesize potential gene expression patterns by interrogating omics data from genomic, epigenomic and transcriptomic levels. Proteomics and metabolomics profiling experiments are then performed to validate how these gene products alter signalling and metabolic pathways. Conventionally, it is believed that integrating as many data types as possible, including mutation, copy number variations/alternations (CNVs/CNAs), DNA methylation, mRNA and miRNA transcriptions, can lead to more robust hypotheses. However, this idea has been challenged by analysing the difference of survival and clinical annotations (e.g., PAM50 subtypes) between clusters made by different combinations of omics data types [38]. The results of this analysis showed that mRNA expression data alone was more indicative for prognostic prediction [28]. Similarly, combining mRNA + miRNA + CNV or mRNA + DNA methylation can improve the accuracy on cancer subtyping for most cancers, compared to using all four omics types together [29]. Furthermore, integrating too many data types can potentially give rise to the ‘curse of dimensionality’, meaning that the sample size is far smaller than the number of variables, potentially leading to overfitting of the model [30]. Finally computational efficiency should be considered when it comes to bioinformatics tools, and it will be jeopardised if training on too much data [28,29]. In conclusion, even without the integration of proteomics and metabolomics data, statistical inference using multi-omics data is currently challenging, and hence better method designs are necessary to overcome these challenges.

## Computational inference approaches for omics data integration

**Figure 2:**
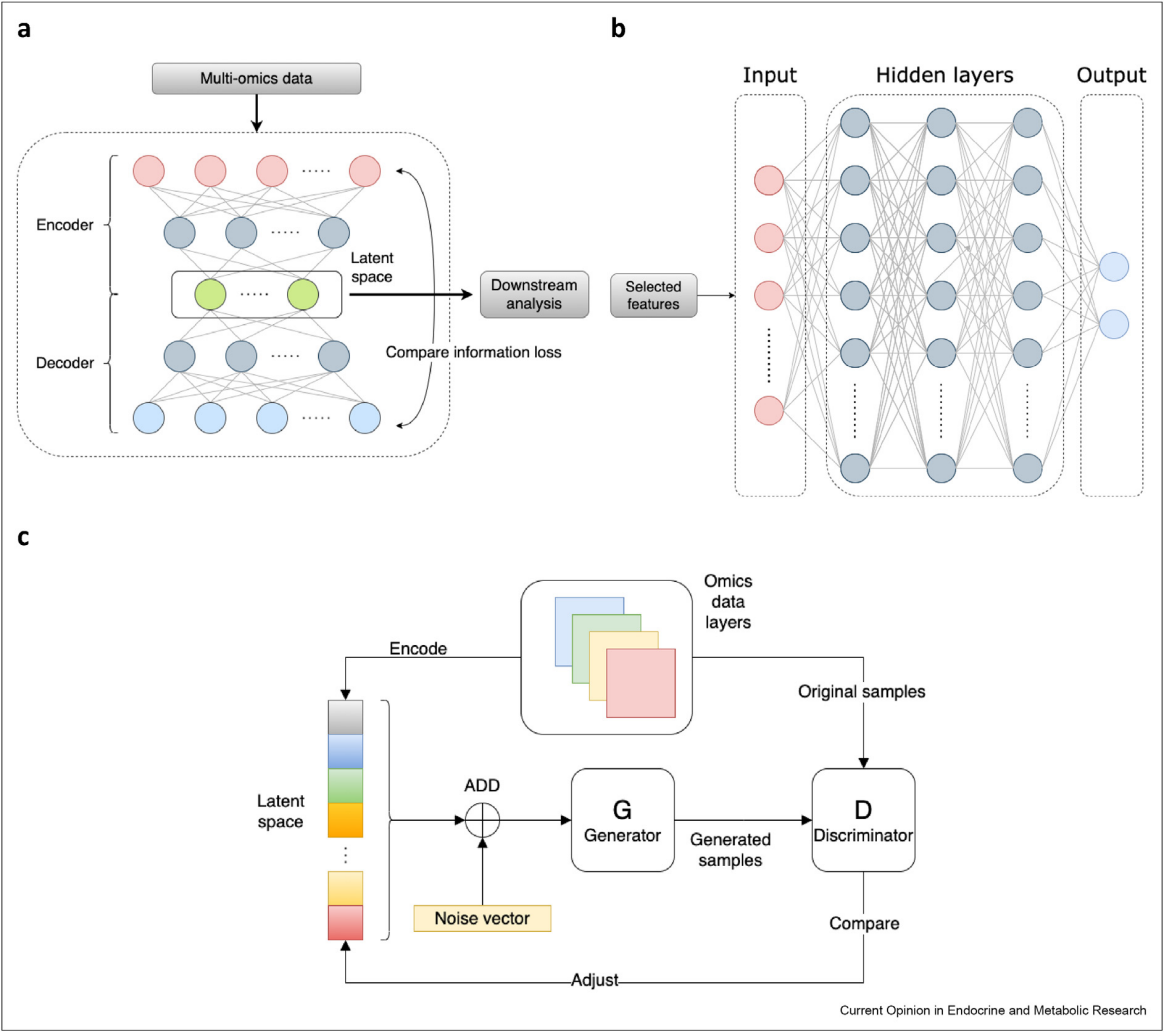
Representation of three common deep learning architectures for multi-omics integration in cancer research. **a)** The autoencoder (AE) architecture composed of an encoder and a decoder. Multi-omics data (inputs) are fed into the encoder to generate the low-dimensional latent space. The latent features are decoded then to reconstruct the original dimension space. The learning process is achieved by minimising the difference between inputs and outputs. **b)** The multilayer perceptron (MPL) architecture for a binary classifier using selected features from multi-omics data to predict a clinical outcome (e.g., metastasis or not). **c)** The generative adversarial network (GAN) adds random noise to latent features and compares generated samples (from the generator) from noise-perturbated features with original samples (by the discriminator). The discriminator then continuously feedbacks to adjust variables in the latent space. In panels **a** and **b**, the nodes, also known as neurons, represent individual data dimensions/features in each layer. The edges connecting these nodes are analogous to the synapses between neurons in biological neural networks: they represent the (weighted) propagation of information between neurons.

Recent computational multi-omics data integration methods for cancer research have focused on utilising deep learning techniques [31, 32, 33∗∗]. Deep learning, also known as deep neural networks, is a category of artificial intelligence techniques that use matrix calculation with nonlinear activation functions (e.g., sigmoid, tanh, rectified linear units [ReLU]) to self-learn the relationship between inputs and outputs [33,34]. These approaches have been piloted to improve the performance of survival analysis, better subtyping, and post-treatment outcome predictions throughout selecting features coalescing expression information at different omics layers. Table 2 lists recent deep learning methods with case studies on breast cancer to achieve different clinical purposes. The major deep learning architectures used in these methods are autoencoder (AE), multilayer perceptron (MLP) and generative adversarial network (GAN) (Figure 2). AE architectures are commonly used for feature selection/dimension reduction for further downstream analysis (Figure 2a). This architecture consists of an encoder, which compresses the original high-dimension inputs to a low-dimension space, known as latent space in machine learning, and a decoder, which reconstructs the original dimension space from compressed features to ensure the minimisation of information loss from the original data. Compared with traditional statistical methods such as non-negative matrix factorisation and canonical correlation analysis, AE enables the approach to learn the nonlinear relationships of different omics layers to contribute to the reduced dimension space [33]. This is a breakthrough because the effect of intra- and inter-omics layers cannot be oversimplified by linear relationships, as molecules are connected by sophisticated networks known as interactomes, such as co-expression networks for intra-layer and gene regulatory networks for inter-layer relationships [35]. MLP architectures are used for supervised auto-classification tasks in which publicly accessible data (e.g., data downloaded from TCGA [23]) are used to train the model and to predict clinical outcomes (outputs) (Figure 2b). The trained model can then be ready to analyse new clinical biopsy profiles and hence generate breast cancer diagnosis and therapy recommendations, as suggested by the MLP models [36, 37, 38∗∗] (Table 2). The GAN architecture was recently implemented in Subtype-GAN [39], which used a similar AE structure to reduce the dimensionality but improved it by adding a discriminator to ensure the robustness of the low-dimensional representations (Figure 2c). This ‘quality control’ was accomplished by mixing latent variables with noise to make sure the low-dimensional representations reflect original inputs even with noise interference [40]. These deep learning methods are promising for multi-omics integration tasks not only because of their ability to construct nonlinear relationships, but also because they can adapt weights and biases by connecting each layer automatically. Traditional statistical methods such as non-negative matrix factorisation often require the manual configuration of large numbers of parameters. This may be error-prone if estimations on parameters are not precise enough, but these can be avoided by deep learning methods as all parameters are adjusted by data feeds in the model [33]. Nevertheless, as these architectures usually consist of numerous hidden layers and nodes, it is difficult to interpret them in clear mathematical formulas, hence making them ‘black boxes’ [33]. Therefore, the introduction of deep learning methods in multi-omics integration studies may lead to novel discoveries in breast cancer biology by their nonlinear and self-adapting abilities. Yet their proper interpretation remains a challenge for future research.

**Table 2:** Deep learning-based multi-omics integration approaches including case studies on breast cancer. Subtype-GAN [39], Denoising autoencoder for accurate CAncer Prognosis prediction (DCAP) [41], DeepProg [42], BRCA Multiomics [38], Multi-Omics Late Integration (MOLI) [43], Survival Analysis Learning with Multi-Omics neural Networks (SALMON) [37], DeepType [36], Concatenation AutoEncoder (ConcatAE) and Cross-modality AutoEncoder (CrossAE) [64], IntegrativeVAEs [65], Drug Response analysis Integrating Multi-omics (DRIM) [66].

Software	Arch.^a^	Purpose	Highlights
Subtype-GAN	GAN	To extract low-dimension features for predicting novel biomarkers and patient stratifications.	The first algorithm to explore the potential of generative adversarial network (GAN) architecture to improve the feature selection process by autoencoder (AE) methods.
DCAP	AE	To predict differentially expressed genes (DEGs) and to discriminate high- and low-risk groups of patients based on predicted DEGs.	Pan-cancer risk prediction system. It ranks the importance of omics data types by mRNA expression > miRNA expression > DNA methylation > copy number variations (CNVs).
DeepProg	AE	To predict patient survival subtypes using supervised machine learning algorithms from reduced dimensions by AE.	Trains on pan-cancer datasets to allow learning from well-established survival of cancer types to predict that for other less-studied cancer types. Flexible using input data types (e.g., mRNA expression).
BRCA Multiomics	MLP	To predict survival and drug responses at the same time by combining two multilayer perceptron (MLP) inferences using survival datasets from TCGA and drug response datasets from GDSC, respectively.	The tool focuses on breast cancer omics data and clinical outcomes and tries to build a connection between patient survival and treatment outcomes to predict if the treatment indeed improves the patient's condition.
MOLI	AE	To predict drug responses from selected features by training on each omics data type separately and then concatenating them into one representation.	MOLI employs a ‘late integration’ strategy and trains on drug response datasets targeting biological pathways rather than specific cancer types to hypothesise other non-traditional drugs for treating BC.
SALMON	MLP	To predict patient survival and characterise which data types are most pivotal predictors by incorporating omics data and clinical annotations (e.g., age).	SALMON groups patients by their ages at diagnosis (young: 26–50, middle: 51–70, elderly: 71–90). It identified that PR status is most predictive for the young group, ER status for the middle group and mRNA co-expression modules for the elderly group.
DeepType	MLP	To extend gene markers (218 DEGs) for breast cancer patient stratification by integrating omics data types and previous PAM50 subtypes.	The first deep learning-based method for patient stratification using mRNA expression only. The involvement of prior knowledge (PAM50 subtypes) addresses de novo clustering problems.
ConcatAE and CrossAE	AE	To question the essence of multi-omics integration, the expression similarity or the difference between omics data types, which is more informative for patient survival prediction.	By comparing learning from the similarity and from the difference between the expression in omics data types, it reports that the expression difference is a stronger predictor.
IntegrativeVAEs	AE	To investigate the inner architectures of AE for feature selection for classifying patient data by clinical annotations (e.g., PAM50 labels and metastasis status).	Patient samples are labelled by distance relapse and the co-effects of gene expression, CNA and clinical annotations are learned by different inner designs of AE for predicting relapse possibilities.
DRIM	AE	To model drug sensitivity from cancer cell lines and drug perturbation by selecting DEGs and analysing them according to pathway enrichment analysis.	DRIM provides a user-friendly website to select drug/cell line of interest for non-experts and allows users to customise the feature selection methods.

a Arch.: The deep learning architecture mainly used in these studies.

One problem targeted by current deep learning approaches is to identify high-risk breast cancer patients using differentially expressed genes (DEGs) that could be experimentally validated, as illustrated by Denoising autoencoder for accurate CAncer Prognosis (DCAP) [41] and DeepProg [42] (Table 2). DCAP discovered nine DEGs to discriminate high- and low-risk groups of breast cancer patients, seven of which (adiponectin, C1Q and collagen domain containing [ADIPOQ], neuropeptide Y receptor Y1 [NPY1R], C–C motif chemokine ligand 19 [CCL19], membrane spanning 4-domains A1 [MS4A1], C–C motif chemokine receptor 7 [CCR7], calmodulin like 5 [CALML5], aldo-keto reductase family 1 member B10 [AKR1B10]) have been already validated to have causal relationships with breast cancer risk in previous studies and two (UL16 binding protein 2 [ULBP2], BLK proto-oncogene, Src family tyrosine kinase [BLK]) were suggested to be associated with breast cancer prognosis [41]. DeepProg reported that high-risk patients can also potentially be predicted by the overexpression of genes from the cell division cycle (CDC) family including CDC20, CDCA8, CDCA5, CDC25C, CDCA2 and the kinesin family member (KIF) such as KIF4A, KIF2C, KIF23, KIF20A, KIF18A, KIFC1, KIF18B, KIF14, and by the downregulation of chromobox 7 (CBX7), enhancer of zeste 1 polycomb repressive complex 2 subunit (EZH1) and multiple genes in zinc finger (ZNF) family (e.g., ZNF18, ZNF540, ZNF589, ZNF554, ZNF763) [42]. AE architectures were applied for feature selection and features extracted by AE methods were generally more accurate for survival prediction compared with other methods [41,42]. Thus, in future multi-omics analysis, it is worthwhile to consider applying AE-based feature selection methods to compress multi-level gene expression (inputs) into stronger predictors (compressed features) which may improve downstream analysis (Figure 2a). Moreover, reducing the omics data to mRNA expression facilitates clinical applicability, as transcriptomics profiling is more widely available in clinical practices (e.g., PAM50) [1,41,42]. We predict that, if there are enough signatures discovered to form a comprehensive patient risk assessment, it may also be possible to apply these deep learning methods on biopsies and assist clinical decisions.

Another problem addressed by deep learning is to model drug responses and predict the long-term post-treatment outcomes. Two representations are BRCA Multiomics [38] and Multi-Omics Late Integration (MOLI) [43] (Table 2). To study drug responses, these two methods both integrated the datasets downloaded from the Genomics of Drug Sensitivity in Cancer (GDSC) database [44] (Table 1), with BRCA Multiomics focusing more on breast cancer drugs while MOLI focusing on pan-cancer drugs [38,43]. The novelties of MOLI manifested in both its integration strategy and training data sources. Firstly, other AE methods usually concatenate different input omics data types together (e.g., an input matrix where rows are samples and columns are features from different omics layers), known as ‘early integration’ in the computational multi-omics modelling field. This integration strategy has several drawbacks including neglecting different distributions in each omics data type by applying the same normalisation strategy and training on too many features without enough samples (i.e., ‘curse of dimensionality’ problem) [28]. MOLI addressed these issues by employing a ‘late integration’ strategy where each omics data type, including somatic mutations, CNAs and mRNA expression, was trained separately to extract features and then integrated into one representation for further classification [43]. Secondly, MOLI applied transfer learning approaches which effectively enlarge the sample size by focusing on a broader question [33,43]. For example, available drug response data for breast cancer are limited, but pan-cancer data which share common pathway regulations with breast cancer can also be used to study potential treatments. In one case study regarding breast cancer treatment, MOLI used pan-drug datasets targeted at the epidermal growth factor receptor (EGFR) pathway for breast, lung, kidney and prostate cancers and discovered that cetuximab and erlotinib may be useful for treating breast cancer [43]. BRCA Multiomics developed two MLP-based classifiers, one for survival prediction (using TCGA datasets [23] from 532 patient samples) and another for drug responses (using GDSC datasets [44] from 42 BRCA cell lines), to predict post-treatment outcomes [38]. By integrating these two classifiers, BRCA Multiomics proposed a framework that analysed survival and drug responses simultaneously using gene expression features to identify if a poor prognosis was caused by intrinsic profiles or treatment responses [38]. The feature selection process was accomplished by neighbourhood component analysis (NCA), a supervised dimension reduction technique, to rank the associations between genes and clinical annotations [38]. Compared with AE methods which select features in an unsupervised manner, NCA improved the clinical relevance but did not have the ability to learn nonlinear relationships. Another multi-omics deep learning study, Survival Analysis Learning with Multi-Omics neural Networks (SALMON) [37], suggest that the age of the patient at time of diagnosis was an important confounding factor regarding patient survival time (Table 2). Therefore, to unite the strengths of both methods, future AE-based methods for feature selection should consider how the architecture can support supervised learning with clinical annotations.

In conclusion, deep learning methods for multi-omics integration have been experimented in a variety of applications to boost our understanding of breast cancer mechanisms, such as selecting expression signatures to characterise the risk of patients (mainly using AE architectures) and building causal relationships between expression profiles and clinical annotations (mainly using MLP architectures). The involvement of GAN also leads us to consider how we can enhance the feature selection procedure. Deep learning inference approaches can exceed other statistical methods in constructing nonlinear relationships, auto-learning and adjustable hidden architectures to fit the scenario of highly heterogeneous multi-omics data effortlessly. Ideally, it is hoped that the highly complex network structures in deep learning approaches will be able to learn the actual causal structure of biological networks from multi-omics data. However, this is currently not the case, as the “black box” problem remains a bottleneck, and hence future computational approaches will need to investigate how the abstract hidden units can be interpreted in the sense of molecular interactions.

## Mechanistic models for drug discovery

Mechanistic modelling, which uses biological hypotheses to build mathematical models and runs bioinformatics algorithms to predict systems behaviour and design experiments for validation, is another widely used approach to identify potential drug targets and drugs that will guide future clinical trials [14,15]. Mechanistic modelling in the context of multi-omics integration covers a wide range of descriptions of biological systems, from dynamic models based on differential equations [45] to network models of molecular and regulatory interactions [46], the common denominator being the fact that the ‘units’ of the model are actual biological entities (e.g., genes, proteins or metabolites) that can be targeted by experimental interventions. This contrast with data inference methods such as the deep learning models presented in the previous section: for example, the inner feature units of a deep learning model are high-level abstractions of data which are difficult to map to concrete entities in biological systems.

One representative approach is proteogenomic analysis, combining next-generation sequencing and mass spectrometry to provide information on functional protein signalling in tumour samples [47]. As proteomics including post-translational modifications (PTMs) and metabolomics data are not as abundant as other omics data types, recent proteogenomic analyses started to combine genomics with a focused analysis of protein kinase and related metabolic activities. The correlation between protein expression and upstream data layers, such as mRNA expression, is not always consistent across the genome. For example, Huang et al. [48] reported positive correlations of CNV, mRNA and protein expression for several key genes of breast cancer cells (especially those involved in metabolic pathways), while Mertins et al. [49] found six genes that are negatively correlated comparing protein with mRNA and CNA. By focusing more on proteomics and metabolomics in multi-omics analysis workflows, more therapeutic targets corresponding to specific protein signalling and metabolic pathways might be discovered.

At the protein level, PTMs have been additionally analysed to unveil breast cancer mechanisms. Krug et al. [47] collected five omics data types using whole exome sequencing, mRNA sequencing, protein, phosphoprotein and acetyl protein expression profiling from 122 breast cancer patient samples and *in silico* analysed differentially expressed metabolic proteins using non-negative matrix factorisation clustering methods. By doing so, they constructed an immune landscape of phospho-retinoblastoma protein (Rb)-dependent kinase activity in TNBC, represented by a higher mammalian target of rapamycin (mTOR) kinase activity accompanied by increasing Rb phosphorylation [47]. In addition, they established that PIK3CA, CDK4/6 and androgen receptor (AR) proteins can be potential therapeutic targets for treating TNBC [47]. By analysing the TCGA BRCA dataset, Lim et al. [50] discovered a previously ignored function of WW domain-binding protein 2 (WBP2) in the TNBC subtype. Then, they validated this hypothesis using *in vitro* cell line models and found that WBP2 is responsible for tumour necrosis factor alpha (TNFα)-induced TNBC cell migration and invasion throughout the ubiquitin-mediated proteasomal degradation of nuclear factor of kappa light polypeptide gene enhancer in B-cells inhibitor, alpha (IκBα), a protein involved in transcriptional regulation by inhibiting nuclear factor kappa-light-chain-enhancer of activated B cells (NF-κB) from binding DNA [50]. Therefore, inhibiting WBP2 can be a potential strategy for treating TNBC [50]. To summarise, proteogenomic analyses on PTMs, including phosphorylation, acetylation and ubiquitinylation, can unravel new molecular determinants of breast cancer; recent developments in mass spectrometry-based data acquisition workflows are an important foundation for these discoveries.

The study of metabolic changes in breast cancer has recently focused on three dysregulated pathways including glucose, amino acid and lipid metabolic pathways [21,27]. Starting from transcriptomic and epigenomic profiling of normal, tumour and residual cells, Radic Shechter et al. [51] discovered that the upregulation of glycolysis and urea secretion can reactivate dormant minimal residual cells causing recurrence and predicted that inhibiting glycolysis may overcome this trend. Indeed, 3-bromopyruvate (3-BP) which inhibits glycolysis can drastically cause residual cell death on breast cancer organoids (patient-derived samples grown in three-dimensional cell culture, which mimic *in vivo* conditions [52]) thus indicating that glycolysis is crucial for breast cancer relapse *ex vivo* [51]. Another metabolic pathway, fatty acid oxidation (FAO), has long been suggested as a potential targetable pathway for breast cancer as surrounding adipose tissues can continuously supply fatty acids into breast tumour cells [21]. Jariwala et al. [53] analysed over 3000 breast tumour samples from TCGA, Molecular Taxonomy of Breast Cancer International Consortium (METABRIC) and CPTAC databases (Table 1) and identified that the dysregulation of FAO can increase CPT1A, an isoform of carnitine palmitoyltransferase I, protein expression and thus proliferation in aggressive HR + breast tumours. Interestingly, they found that ranolazine, an FAO inhibitor which is previously used for treating hearted related chest pain, can also be used to inhibit breast tumour proliferation according to their ranolazine-treated breast cancer xenograft models (injection of patient cells into nude mice [54]) [53]. Gong et al. [55] first identified the positive correlation between mRNA and protein expression of metabolic genes involved in 465 TNBC patient samples and performed metabolic pathways enrichment analysis to cluster samples into three metabolic-pathway-based subtypes (MPSs), with MPS1 (lipogenic subtype) represented by upregulation of lipid metabolism and MPS2 (glycolytic subtype) by upregulation of carbohydrate and nucleotide metabolism. Metabolic inhibitors were assessed by *in vitro* models (cell lines) to suggest lipid synthesis inhibitors for MPS1 subtype and glycolysis inhibitors for MPS2 subtype [55]. They also validated that lactate dehydrogenase (LDH) inhibitors might sensitise MPS2-type TNBC to immunotherapy (e.g., anti-PD-L1) by *in vivo* experiments [55].

In conclusion, several recent pieces of evidence suggest that breast cancer can be considered as a metabolic disease as well as a genomic disease. Besides, as metabolic inhibitors often will not negatively affect normal cells [51], they are more likely to be specific for the cancer cells and thus help to maintain the quality of life for breast cancer patients. With the development of experimental protocols of proteomics and metabolomics profiling, more therapeutic options have been proposed for targeting signalling kinases and metabolic pathways. A more comprehensive network of breast cancer mechanisms is being identified by integrating these two omics layers with other omics data types, such as mRNA expression. Although the causal links between proteomics and other omics layers are complex, metabolic genes have been reported to be positively correlated with mRNA expression in a few studies [48,49,55], suggesting the alternative use of transcriptomics data to infer metabolic pathways. In addition, protein acetylation has also been used to measure cellular metabolism [47]. We envisage that future multi-omics mechanistic integration will focus more on proteomic and metabolic analyses as well as their correlation with upstream omics layers to build a comprehensive multi-omics interactive network from genotype to phenotype and corresponding personalised treatment.

## Future directions

Multi-omics integration for breast cancer modelling has drawn considerable attention. This approach has been driven by developments in diverse disciplines – including molecular biology, biochemistry, bioinformatics and computer science – to discover novel mechanisms and ultimately contribute to clinical precision oncology. Despite current achievements in various cancer- and drug-specific data programs, integration algorithms and proteogenomic workflows, there are a variety of ongoing questions for future investigation. For instance, data are not available in equal amounts across all omics layers, where genomic, epigenomic and transcriptomic data are enriched, while proteomic and metabolic data are much scarcer. This requires the research community to reduce this gap to enable the construction of global patterns of information flow from genotype to phenotype.

Two very different but complementary types of modelling approaches are contributing to our understanding of multi-omics data: 1) the statistical modelling at the heart of deep learning and computational inference, which focuses on the identification of predictive ‘features’ that identify, for example, breast cancer subtypes or predict treatment outcomes, and 2) mechanistic modelling, often based on systems of differential equations or network descriptions of cellular pathways, which serve to describe and simulate the dynamic function of biological systems at the molecular level. The deep neural networks used in statistical models for computational inference typically lack proper interpretability: the hidden units of these models represent high-level abstractions based on the combination of a variety of data (e.g., expressions of different genes or gene products). Their interpretation requires a mapping of this abstract information onto the actual molecular networks which are the centrepiece of mechanistic models [56]. Ideally, the molecular features selected as predictive by a deep learning algorithm can be mapped onto the network of molecular interactions represented by a comprehensive mechanistic model of cancer biology. This will allow moving from the prediction of outcome or patient status to an active intervention strategy targeting the specific cellular pathways underlying a disease phenotype. Too often, these two modelling approaches are developed independently by investigators in the areas of computer sciences and biological sciences, respectively; in the future, it will be important to establish closer interdisciplinary communication opportunities and collaborations to bridge such gaps.

In practice, clinical applications such as a personalised drug recommendation system, would benefit from single-omics tests and a small number of biomarkers. Nevertheless, our understanding of breast cancer mechanisms is still in the phase of discovery, where a larger number of druggable targets, as well as a comprehensive understanding of their embedding in functional pathways across all omics levels are of critical importance. Therefore, we need multi-omics modelling to understand the complex molecular network landscape of breast cancer and maximise our chances to develop efficient applications for precision medicine.

## Authors contributions

HM wrote the manuscript; RB, CF and JMS conceived the project and edited the manuscript. All authors have read and approved the final version.
